# Effectiveness of infertility counseling on pregnancy rate in infertile patients undergoing assisted reproductive technologies: A systematic review and meta-analysis

**Published:** 2017-07

**Authors:** Nahid Maleki-Saghooni, Malihe Amirian, Ramin Sadeghi, Robab Latifnejad Roudsari

**Affiliations:** 1 *Student Research Committee, Department of Midwifery, School of Nursing and Midwifery, Mashhad University of Medical Sciences, Mashhad, Iran.*; 2 *Department of Obstetrics and Gynecology, School of Medicine, Mashhad University of Medical Sciences, Mashhad, Iran.*; 3 *Nuclear Medicine Research Center, Mashhad University of Medical Sciences, Mashhad, Iran.*; 4 *Evidence-Based Care Research Center, Department of Midwifery, School of Nursing and Midwifery, Mashhad University of Medical Sciences, Mashhad, Iran.*

**Keywords:** Counseling assisted reproductive techniques, Infertility, Meta-analysis, Pregnancy rates

## Abstract

**Background:**

Psychological interventions such as counseling for infertile patients seem to increase pregnancy rate.

**Objective:**

The aim of this systematic review and meta-analysis was to examine if counseling improves pregnancy rate among infertile patients. Thus, randomized controlled trials investigating the effect of counseling on pregnancy rate in infertile patients undergoing ART were pooled in a meta-analysis.

**Materials and Methods:**

The databases of PubMed, Scopus, Cochrane, Google Scholar, and Persian databases including SID, Iran Medex, and Magiran were searched from 1997 to July 2016 to identify relevant articles. Included studies were trials on infertile patients (women or couples) receiving counseling independent of actual medical treatment. The outcome measure was pregnancy rate. Out of 620 relevant published trials, a total of nine RCTs were ultimately reviewed systematically and included in a meta-analysis to measure the efficacy of counseling on pregnancy rate. Odds ratio and Risk difference were calculated for pregnancy rate. All statistical analyses were done by Comprehensive Meta-analysis Version 2.

**Results:**

Nine RCTs involving 1079 infertile women/couples were included in the study. The findings from RCTs indicated significant effect of counseling on pregnancy rate so that there was a positive impact of counseling on pregnancy rate (OR= 3.852; 95% CI: 2.492-5.956; p=0.00) and (RD= 0.282; 95%; CI: 0.208-0.355; p=0.00).

**Conclusion:**

Counseling was found to improve patients’ chances of becoming pregnant. So counseling represents an attractive treatment option, in particular, for infertile patients who are not receiving medical treatments.

## Introduction

I achieve a pregnancy following one year of sexual relationships without conception (1). Around 10-15% of couples experience infertility that influences equal numbers of men and women (2, 3). The etiology of infertility is related to a female factor in 40% and male factor in another 40%. In 10-20% of the cases, both male and female factors are involved. Unexplained infertility is observed in up to 10% of the cases (4). Couples often describe the experience of infertility as a critical, significant life event (5). So rather than a medical issue, infertility may be depicted as a source of anxiety, fear, sadness, and frustration (6-8). Couples who suffer from fertility issues often use assisted reproductive technologies (ART) to realize their wish to have children (9). Also, there is increasing evidence that psychological factors, are related to ART and its outcome and success (10, 11). So it is widely accepted that experience of infertility and decision to engage with ART services as a patient, can entail wide-ranging and long-lasting psychosocial impacts (6). But the effect of psychological symptoms, on fertilities, remained controversial. Although it is generally accepted that infertility causes significant levels of psychological distress, the possibility that distress contribute to infertility remains a topic of debate (12). 

Several investigations have shown that psychological issues have a detrimental effect on fertility and their reduction is connected with an increase in pregnancy rate (13-15). Klonoff-Cohen *et al* and Smeenk and colleagues reported the raised anxiety and depression may cause lower pregnancy rate (11, 16). One way to help infertile couples is to use standard psychological interventions like counseling (17). Since 1980s, the need for psychosocial counseling, provided by a skilled specialist professional to guarantee comprehensive holistic care in ART, began to be recognized (18). Latifnejad Roudsari and Allan argued, as infertility is a multifaceted problem, professionals who are working with infertile couples require employing holistic approaches such as counseling in which all psychological, social and cultural needs of individuals be considered (19).

Infertility counseling is proposed as an integral part of a multidisciplinary approach. It has been strongly recommended by various governmental, medical and community associations in order to help infertile people (20). In recent years, infertility counseling has become a specialist form of counseling requiring professional expertise and qualifications (21). In this context, there are some studies that investigate the effect of counseling on pregnancy rate including Li and colleagues reported the experimental group had higher pregnancy rate than the control group (22). Gorayeb *et al* found participants in the psychological intervention group obtained pregnancy rates of 39.8% vs. 23.2% in nonparticipants which was significantly higher (23).

In another study by Chan and colleagues, it was suggested that the intervention and control group did not differ significantly in the pregnancy rate and the trend was non-significant (p=0.065) (24). Although a contradiction is seen between the results of these studies but Frederiksen *et al* in a meta-analysis assessed the efficacy of psychosocial interventions for psychological and pregnancy outcomes and showed that psychosocial interventions increase the chance of pregnancy (25). However, it should be taken into account that this meta-analysis had several limitations such as lack of focus on counseling alone, the high level of heterogeneity as well as a number of non-controlled trials. In order to arrive into a valid conclusion, it is essential to compare the resultant pregnancy rate solely from controlled studies and to the best of our knowledge, the efficacy of infertility counseling on pregnancy rate in infertile women has not been systematically reviewed. So the aim of this systematic review and meta-analysis was to assess the effectiveness of infertility counseling on pregnancy rate in infertile women undergoing assisted reproductive technologies.

## Material and Methods

**Data sources and search strategy**

In this study, a systematic literature review was conducted using the following electronic databases as the most appropriate resources to identify published studies: PubMed, Scopus, Cochrane, Google Scholar, and Persian databases including SID, Iran Medex, and Magiran using equivalent keywords until July 2016. Search keywords were (assisted reproductive technologies or ART) AND (IVF, ICSI, ET, IUI) AND (infertility OR sterility) AND (counseling, cognitive behavioral counselling, cognitive behavioral treatment, nursing crisis intervention, psychological interventions, mind/body intervention, psychiatric intervention, psychosocial counselling, cognitive behavioral intervention) in the title, abstract, or keywords. 

**Figure 1:**
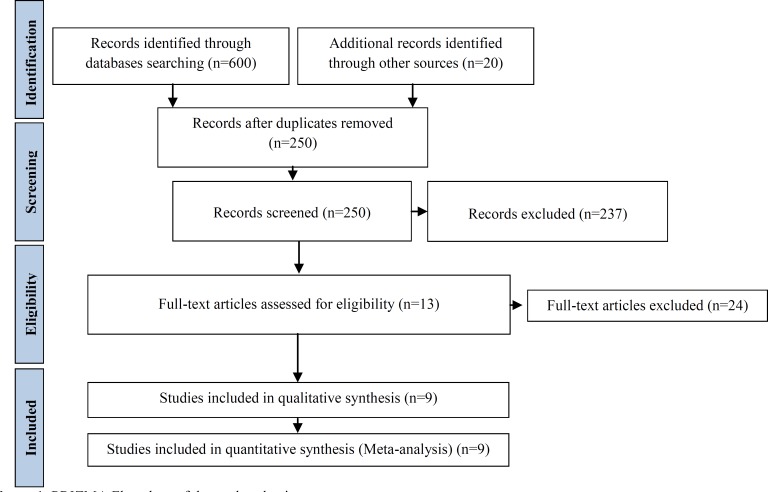
PRIZMA Flowchart of the study selection process

All of the aforementioned databases were searched for published (randomized controlled trials, RCTs) in both English and Persian languages. Many of the search attempts generated duplicate articles, or articles unrelated to the study, which were not considered in the reviews. In addition, reference section of the relevant trials, systematic reviews, and meta-analyses were manually checked to identify further trials missed by the electronic search. In the process of extraction, one of the investigators reviewed both the title of the articles and the abstract to determine its suitability for inclusion. The process of the search and selection of RCTs is shown in figure 1.

The selected articles had to meet the following criteria to be included in the review:

**Inclusion criteria**

Study participants had to be infertile women and men or infertile women only. They must be undergoing treatment for assisted reproductive technologies such as (IVF: in vitro fertilization, ICSI: Intra-cytoplasmic sperm injection, ET: embryo transfer and IUI: intra uterine insemination). Different definitions and classifications of infertility exist, however for clinical purposes, the gold standard definition of infertility, i.e. ‘the inability of a couple to achieve conception or to bring a pregnancy to term after a year of regular, unprotected sexual intercourse, was considered (26). Since there is no clear, accepted definition used by everyone, the present meta-analysis included any studies using a sample labeled as infertile according to the above-mentioned definition. The control groups did not receive any counseling services. They were either on waiting lists or received routine care.

RCTs studies were included, in both English and Persian languages. The included studies retrieved from published sources, and the literature search was not limited to any timeframe. Interventions consisted of counseling as a psychological face-to-face intervention: (i) designed to influence psychological functioning; and (ii) incorporating psychological strategies through interaction. The counseling could be provided using different methods (individual, couple or group) in a variety of settings.

**Outcome measure**

The outcome measure in this study was pregnancy rate, which was measured through β-human chorionic gonadotropin test (β- hCG), sonography or both of them.

**Study selection and data extraction**

The study selection and data extraction were undertaken by two independent reviewers. According to search results, authors scanned the identified results, abstracts, and relevant records. Full articles of all potentially relevant trials were retrieved. All retrieved studies were scrutinized to check for multiple publications of the same trials. For each study, we extracted the following data according to a pre-defined checklist including first author, year and the location of the study, study design, participant, intervention, comparison, dropout, tools, outcome, and quality of trials. Data were independently assessed by two reviewers and disparities were resolved by discussion with a third researcher. Overall, there was complete agreement between the two reviewers. The summarized characteristics of the included studies are shown in table I.

**Quality assessment of the included studies**

**Figure 2:**
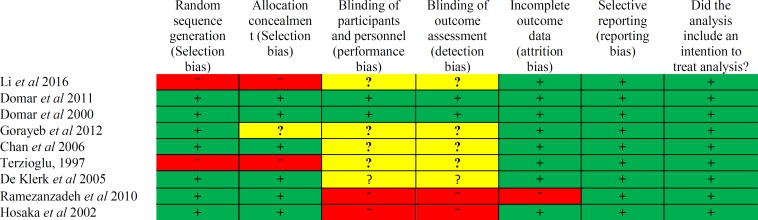
Risk of bias summary: Systematic review. Author’s judgments of risk of bias item for each included study

**Figure 3:**
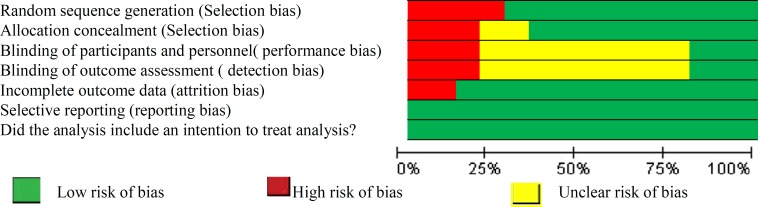
Risk of bias graph: Systematic review. Author’s judgments of risk of bias presented as percentages across all included studies

The quality of the included studies was evaluated by Oxford Center for Evidence Based Medicine checklist for RCTs. This tool has been designed in two sections to assess internal and external validity. Internal validity in this review was checked by six general questions including the way of patient's assignment, similarity, and matching of groups, equality of allocated treatment, losses to follow-up and intention-to-treat analysis, blindness and effect size which was assessed with three answers of yes, no and unclear. Risks of bias assessment are shown in figure 2, 3.

**Statistical analysis**

We interpreted the results using two effect sizes, Odds ratio (OR) and risk difference (RD). For heterogeneity evaluation, Cochrane Q test (p<0.05 as statistically significant) and I2 index were used. According to studies difference, random effects models of analysis were used (p=0.462; I^2^=0%). The pooled estimates of OR and RD of pregnancy rate were calculated. The latter was used to assess how much of the variance across studies is likely to be real and is not due to sampling errors. For publication bias evaluation, funnel plots were used. To quantify the asymmetry in the plots, Egger’s regression intercept was used (Table II). All statistical analyses were done by Comprehensive Meta-analysis Version 2 (Biostat, Englewood, NJ, USA).

## Results

A total of nine RCTs met the inclusion criteria and were included in the meta-analysis (Table I). All of the nine articles were randomized controlled trials designed to evaluate the efficacy of counseling for infertile patients. In total 481 patients were assigned to the intervention group and 598 patients were assigned to the control group. Out of nine studies, two were carried out in Europe (Netherlands, Israel), five in Asia (two in China, Iran, Japan, and Turkey one each), one in Brazil and another one in the United States of America. 

All of the studies were published in English. Further, all of the studies reported on the patients receiving ARTs including IVF, ICSI, and ET or receiving other medical treatments like IUI for infertility. The mean duration of infertility was 10.34 yr (2-18.68). The mean age of the participants was 31 yr (26-36). A total of five studies included only infertile women and four studies included infertile couples. The randomized allocation was used in all of the studies. Outcome measured included: quality of life, anxiety, childbearing importance, depression, life satisfaction level NK-cell activity and pregnancy rate was reported in all of the studies and it was considered as the main outcome in this study (22, 24, 27-30) 

Most of the studies measured outcomes between 2 wk to 24 months after the intervention. The intervention strategy employed included counseling. The duration of each session of intervention was varied between 1-2.5 hr. The number of sessions was from 3-10 times a week. In two studies, patients in the control group did not receive any specific intervention and in seven studies, the control group received routine care or same psychological treatment. The study quality of all nine studies was thoroughly assessed using Oxford Center for Evidence Based Medicine checklist for RCTs. 

All of nine studies reported measured pregnancy rate with β-human chorionic gonadotropin test (4 studies), sonography (2 studies) or both (3 studies) )Table I). Only two studies used a blind assessment of outcome. In all of the studies, between the intervention and control group, there were no statistically significant differences in relation to social demographic and clinical characteristics. Also, there was no significant difference between two groups concerning pregnancy rate at baseline. There were three studies that reported less than 20% participant dropouts during the course of study, while six studies had 32-47% dropouts. Inclusion criteria were described in five studies, whereas four studies did not report such criteria exactly (Table I). 

In the reviewed studies counseling provided with various methods, for example Li *et al*, used Mindfulness-Based Intervention as psychosocial intervention that incorporates the following approaches to cultivating mindfulness: mindfulness of thoughts and feelings, mindfulness of the body through guided body scan, body awareness meditation, mindfulness of attitudes and waving the mindfulness practices into the daily life (22). Two trials applied Mind/ Body Program as an effective stress-management approach that its focus is on cognitive behavior therapy, relaxation training, negative health behavior modification, and social support components (24, 31). 

Two trials introduced counseling as psychological intervention or cognitive-behavioral therapy in which clients receive relaxation training, cognitive restructuring, methods for emotional expression, and nutrition and exercise information techniques of stress control (30, 32). Hosaka *et al* used problem-solving and psychological support as a psychiatric group intervention (28). Two trials applied counseling and explained that it has two main elements of support and giving information and helps clients to improve their adaptation to the treatment and their ability to cope with problems associated with the treatment, concurrent with a decrease in stress level (27, 29). 

All these studies acknowledged that counseling has affected through Stress reduction mechanism. Since distress significantly reduced the probability of conception we pooled these studies together if counseling has the possible decreasing impact on stress and increase the possible chance of pregnancy. In this study, meta-analysis was performed based on both OR and RD. At first, the results of the meta-analysis are reported based on the OR. Nine trials assessed the effect of counseling on pregnancy rate in infertile couples (22-24, 27-32). Heterogeneity was examined with Chi-square test and the result showed that the difference between the results of studies was low, which means that, the studies were homogeneous (p=0.462; I^2^=0%). 

**Figure 4:**
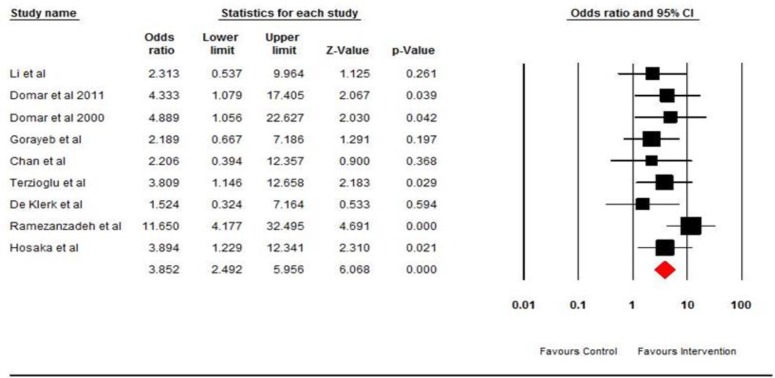
The effect of counseling on pregnancy rate based on Odds ratio. The horizontal lines denote the 95% CI, ■ point estimate (size of the square corresponds to its weight); ◊ combined overall effect of treatment

The result of nine pooled studies showed that pregnancy rate has an increase in patients receiving counseling as compared to the control groups (OR 3.852 95%; CI 2.492-5.956; p=0.00) that indicating a statistically significant effect for counseling with respect to pregnancy rate. The forest plot is shown in figure 4. Use of a funnel plot revealed unsymmetrical results with respect to pregnancy rate. These results can be interpreted as a hint of possible publication bias, as smaller studies that did not demonstrate efficacy might not have been published. The results of the meta-analysis based on RD showed that nine trials assessed the effect of counseling on pregnancy rate in infertile couples (22-24, 27-32). Heterogeneity was examined with Chi-square test and the result showed that the studies are homogeneous (p=0.499; I^2^=0%). 

**Figure 5:**
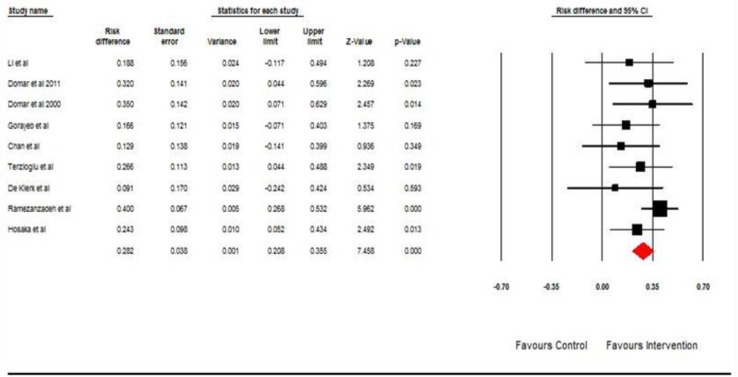
The effect of counseling on pregnancy rate based on risk differences. The horizontal lines denote the 95% CI, ■ point estimate (size of the square corresponds to its weight); ◊ combined overall effect of treatment**.**

The result of nine pooled studies showed that pregnancy rate has an increase in patients receiving counseling as compared to the control group (RD 0.282 95%; CI 0.208-0.355; p=0.00). Compared with the previous meta-analysis, based on OR this analysis also showed a statistically significant effect for counseling with respect to pregnancy rate. The forest plot is shown in figure 5. Also, funnel plot revealed unsymmetrical results with respect to pregnancy rate as reported to the previous plot that can be interpreted as a hint of possible publication bias.

**Table I:** Characteristics of 9 clinical trials included in study

**Author ** **Year** **Location of the study**	**Design**	**Participants**	**Intervention**	**Comparison**	**Dropout rate**	**Tool**	**Outcome**	**Quality indicators**
Li *et al*. 2016 China (22)	Non- randomized controlled study	Infertile women N=166	Mindfulness-based intervention 6 week, 2-2.5 hr. per week N=58	Routine care N=50	Attrition rates=58 35%	Demographic information SCScale Ch-FFMQ Questionnaire FertiQoL COMPI clinical or ultrasound parameters	Significant increase in mindfulness, self-compassion, meaning-based coping strategies and all FertiQoL domains also statistically significant differences between participants in the pregnancy rates was found.	-/+/+/+/-/+
Domar *et al*.2011 Boston (31)	RCT	Infertile women N=143	Ten-session mind/body program every 6-8 weeks N=46	Routine care and receive a $50 spa gift certificate for every 3 months N=51	46 withdrew 32%	Demographic form β-hCG levels sonography	Pregnancy rates did not differ between the two groups for cycle 1; however, a difference was seen for cycle 2 The analyses confirmed the higher Pregnancy rates in the MB patients during cycle 2.	+/+/+/+/+/+
Domar *et al*. 2000 Israel (32)	RCT	infertile women N=184	cognitive-behavioral met for 2 hr on a weekly basis for 10 weeks n = 47	Support spent the 1st hr of each session n =48 routine-care control group n = 25	N=64 34%	Demographic characteristics monthly fertility medication and treatment diaries Viable pregnancy was a live birth.	Women who participated in one of two group psychological interventions experienced significant improvements on a number of psychological measures as they continued to attempt conception. These differences were not due to any initial group differences on demographic or medical treatment parameters. Group psychological interventions appear to lead to increased pregnancy rates in infertile women.	+/+/+/+/+/+
Gorayeb *et al*. (2012) Brazil (23)	RCT	Infertile couples N=285	N=93 cognitive behavioral intervention consisted of five weekly meetings lasting two hours each	N=95 received no intervention only received medical intervention	N=97 34%	Clinical pregnancy by ultrasound examination	Patients receiving the psychological intervention presented higher pregnancy rates (IG=39.8%) when compared to the control group (CG=23.2%), (χ^2^=6.03, p=0.01, OR of 2.2 (CI: 1.16-4.13).	-/+/+/+/-/+
Chan *et al.* (2006), China (24)	RCT	Infertile women N=227	N= 69, received four sessions of EBMS group counseling	N= 115, did not receive any intervention	N=43 19%	State-Trait Anxiety Inventory. Childbearing importance index. clinical profile	There was a significant drop of State Anxiety score in the intervention group ^2^analysis suggested a marginally higher pregnancy rate in the intervention group than in the control group, although the trend was non-significant (p=0.065).	+/+/+/+/-/+
Terzioglu, (1997) Turkey (29)	Experimental study	Infertile Couples N=90	N=30 the experimental group was given counseling and were informed and supported throughout the steps of the ART	N=30 The control group followed the normal procedure of the unit	N=30 33%	STAI BDI LSI Patient follow-up chart β-human chorionic gonadotropin test fetal heartbeat observed on ultrasonography	Couples in the experimental group had lower anxiety and depression scores than the couples in the control group. Life satisfaction scores and pregnancy rates were higher for couples in the experimental group than for the couples in the control group. Statistical evaluation showed that the difference between the experimental group and the control group was significant (p<0.05).	-/+/+/-/-/+
De Klerk* et al*. (2005), Netherlands (27)	RCT	N=84 infertile couples	N=22 received three counselling sessions, each of ,1 h duration: one before, one during and one after the first IVF cycle	N=22 routine-care	N=40 47%	DRK HADS Pregnancy test	No differences between the intervention and control groups were found on the depression subscale and on the anxiety subscale	+/+/+/+/-/+
Ramezanzadeh *et al*. (2010), Iran (30)	RCT	N=140 infertile couples	N=70 Cognitive behavioral therapy (CBT) and 20 to 60 mg per day of fluoxetine	N=70 same psychological treatment	N=0	Demographic-social questionnaires BDI SRRS blood hormones tests	Intervention in the treatment group significantly decreased the depression score pregnancy occurred in 33 (47.1%) couples in the treatment group and in only 5 (7.1%) couples in the control group. There was a significant difference in pregnancy rate between the treatment and control groups (*χ*^2^=28.318, p<0.001).	+/+/+/-/-/+
Hosaka et al., Japan (28)	RCT	N=80 Japanese infertile women	N=37 intervention program consists of 5 weekly 90-min sessions led by two professionals such as a psychiatrist and a nurse	N=37 standard treatment	N=6 7.5%	POMS HADS plasma samples	The pregnancy rate in the intervention group (14/37, 34.8%) was significantly higher than that of the control group (5/37, 13.5%, test, p=0.016).	+/+/+/+/-/+

Quality indicators includes: Assignment, groups similarity, groups treated equally, losses to follow-up, blindness, what were the results

FertiQoL: Fertility quality of life

MB: Mind/body.

IG: intervention group

CG: control group

CI: confidence interval

SCS: Self-Compassion Scale

OR: Odds ratio

BDI: Beck’s Depression Inventory

DERS: Difficulties in Emotion Regulation Scale

Ch-FFMQ: The Chinese version of Five Facet Mindfulness Questionnaire

EBMS: Eastern Body-Mind-Spirit

COMPI: The Copenhagen Multi-Centre Psychosocial Infertility

STAI: Spielberg's State and Trait Anxiety

LSI: Life Satisfaction Inventory

DRK: Daily Record Keeping Chart

HADS: Hospital Anxiety and Depression Scale

SRRS: Social Readjustment Rating Scale

POMS: Profile of mood states

**Table II:** Egger’s regression intercept for publication bias evaluation.

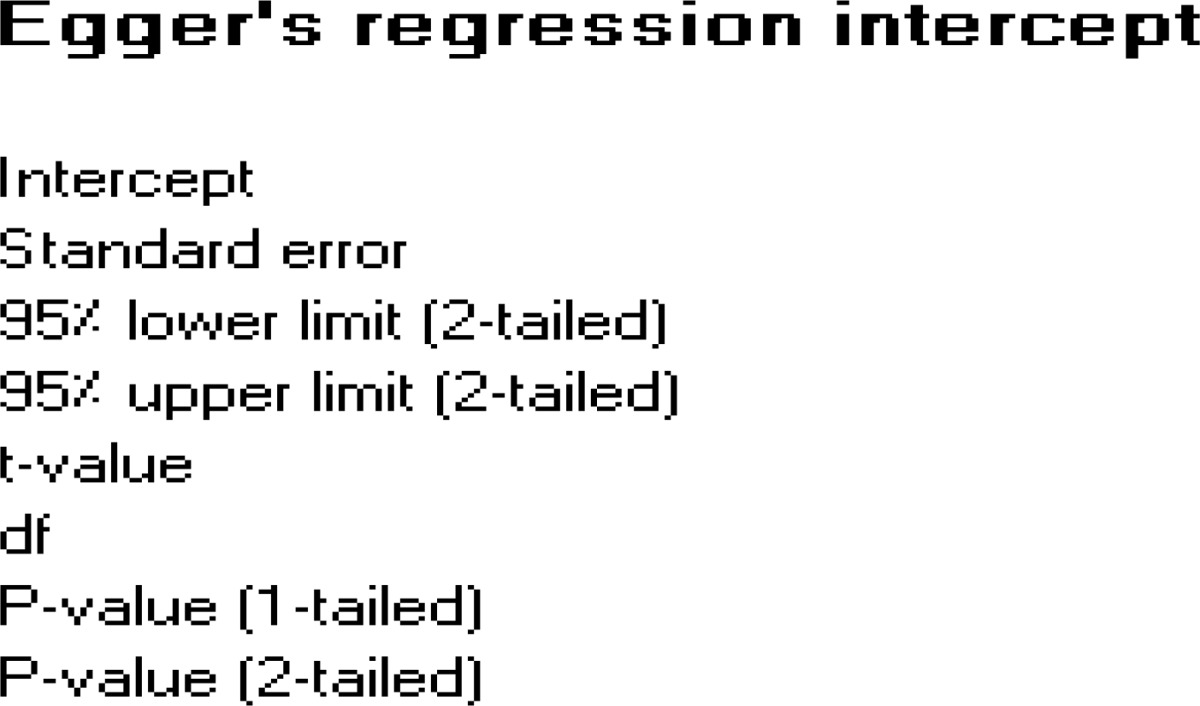

## Discussion

In this research, all studies acknowledged that counseling has affected through stress reduction mechanism. Because distress significantly reduce the probability of conception so counseling has the possible decreasing impact on stress and increase the possible chance of pregnancy and more reviews along with the results of this meta-analysis demonstrate the efficacy of counseling for patients suffering from infertility with respect to pregnancy rate. This meta-analysis’ findings that is pointing to an increased pregnancy rate due to counseling are in line with the results of Boivin and de Liz and Strauss that investigated the efficacy of psychological interventions for infertile patients (20, 33). Also, the results of one systematic review and meta-analysis by Hammerli *et al* that analyzed the efficacy of psychological interventions for infertile patients indicated that psychological interventions improve some patient's chances of becoming pregnant and it has a critical impact on pregnancy rate (34). This significant effect of psychological interventions was only discovered for couples who not received ARTs.

Although the result of this review is in line with our study, it should be noted that in Hammerli *et al* study infertile couples who were not undergoing ART, were investigated, and based on numerous studies, stress levels between the two groups were not the same and in ART group was higher (35-38). So the results should be used with caution to be generalized. In this study psychological interventions consisted of counseling, educational interventions, relaxation and psychodynamic/ analytic interventions (34). The positive effect of such interventions on pregnancy rates must be interpreted cautiously because a clear explanation for this effect cannot be provided (34). Based on the results of some studies one possible explanation for couple's increased rate of pregnancy following psychological interventions such as counseling could be the effectiveness of these interventions in reducing stress and anxiety because several studies have addressed the relationship between these two factors and pregnancy rate so that high levels of depressive symptoms, anxiety, and distress have been associated with reduced chances of becoming pregnant (10, 11, 13, 16, 41, 39, 40).

Another explanation for couple's increased rate of pregnancy could be that sexual activity is disrupted -at least temporarily- in more than half of the couples suffering from infertility and psychological interventions may positively impact on sexual behavior, and thus increase couples chances of becoming pregnant on this basis alone (42). In spite sexual behavior was not assessed in the present review, Boivin has found that all the relevant psychological intervention studies that evaluated couple's sexual behavior reported a positive impact with respect to their frequency of sexual intercourse (20). Such increased rate of sexual intercourse following psychological interventions may provide a link to the increased rate of pregnancy (20, 43).

In order to arrive at a definitive conclusion, infertile patient's sexual behavior and their mental distress would have to be evaluated simultaneously to determine their relative impact on the pregnancy rate thus; future investigations should examine the relationship between couple's frequency of sexual activity and sexual satisfaction with pregnancy rates. A further illustration for increase in pregnancy rate among infertile patients following psychological interventions may be the dropout rate in ARTs. The overall success of ART in terms of cumulative pregnancy rate is strongly impacted by early cessation of treatment. High levels of anxiety and depression in infertile patients and the use of a standard treatment strategy for IVF and ICSI are connected with a higher dropout rate (44, 45). 

There are a few limitations suggested for the present study results. One limitation of this study is the small number of articles but these studies were found through searching several relevant databases and so that it seems that no related article has been missed. Additionally, the funnel plots point to possible publication bias. It is possible that other smaller, relevant studies that failed to prove an effect for counseling simply have not been published. The present findings are also limited by the fact that only nine randomized controlled trials were found as a result of the literature search. The studies also differed strongly with respect to the time period at which assessment occurred, ranging from 2 weeks to 24 months post-intervention. The present study does not provide any information on whether the patients differing in cause and duration of infertility may benefit more or less from counseling. The results of the present meta-analysis can only provide answer regarding the efficacy of counseling for infertile patients with respect to pregnancy rate.

**Recommendations for future research**

The present meta-analysis measured the efficacy of counseling for infertile patients in connection with pregnancy rates. Future research could investigate the impact of different types of psychological interventions, in terms of the target people (single, couple or group), the type (e.g. psychotherapy, counseling, and education) and the intensity (frequency and duration) of the interventions in randomized trials. Furthermore, the level of mental distress is important covariates in experimental studies when trying to arrive at conclusions regarding the differential effects of psychological treatments in specific populations. Study results are still inconclusive regarding the best way for counseling aimed at infertile patients. Although De Liz and Strauss have found group and individual/couple interventions to be similarly effective, Boivin reports of group interventions being more effective, Boivin reports of group interventions being more effective (20, 33).

Future research should also further examine the gender specific effects of counseling for infertile patients. Women and men ought to be analyzed separately as there are important differences in their processing of fertility-related information. In addition, infertility patients’ level of mental distress often varies according to the phase of infertility in which they find themselves. Therefore, patients’ phase of infertility must also be taken into account. Additionally, the dose-response relationship needs to be assessed further by focusing on the duration and intensity of counseling. 

Overall, there are methodological flaws present in some primary studies. It is imperative that future research studies employ high-quality research design and hat their results be presented in a clear, unequivocal manner in order to achieve more definitive conclusions regarding the efficacy of counseling for infertile patients.

**Clinical implications**

Counseling should be integrated in the treatment of infertility as the present study indicates that counseling is effective in increasing pregnancy rate. 

## Conclusion

The findings of the present study provide some evidence in support of integrating counseling as an early remedial strategy for infertile patients. Counseling appears to increase infertile women’s chances of becoming pregnant. On the basis of the results, counseling is beneficial for infertile patients, but more randomized controlled trials are needed**.**
